# Evaluating Nanofiltration and Reverse Osmosis Membranes for Pharmaceutically Active Compounds Removal: A Solution Diffusion Model Approach

**DOI:** 10.3390/membranes14120250

**Published:** 2024-11-25

**Authors:** Yonghyun Shin, Tae-Mun Hwang, Sook-Hyun Nam, Eunju Kim, JeongBeen Park, Yong-Jun Choi, Homin Kye, Jae-Wuk Koo

**Affiliations:** 1 Department of Environmental Research, Korea Institute of Civil Engineering and Building Technology, 283 Goyangdae-ro, Ilsan-gu, Goyang-si 10223, Republic of Korea; shinyonghyun@kict.re.kr (Y.S.); taemun@kict.re.kr (T.-M.H.); fpnsh@kict.re.kr (S.-H.N.); kej@kict.re.kr (E.K.); jbpark@kict.re.kr (J.P.); hominkye@kict.re.kr (H.K.); 2 Civil and Environmental Engineering, Korea University of Science & Technology, 217 Gajung-to Yuseong-gu, Daejeon 34113, Republic of Korea; 3 Civil and Environmental Engineering, Kookmin University, 77 Jeongneung-ro, Seongbuk-gu, Seoul 02707, Republic of Korea; choiyj1041@gmail.com

**Keywords:** trace organic contaminants (TrOCs), pharmaceutically active compounds (PhACs), reverse osmosis (RO), nanofiltration (NF), solution diffusion model

## Abstract

Trace organic contaminants (TrOCs), including pharmaceutically active compounds (PhACs), present significant challenges for conventional water treatment processes and pose potential risks to environmental and human health. To address these issues, nanofiltration (NF) and reverse osmosis (RO) membrane technologies have gained attention. This study aims to evaluate the performance of NF and RO membranes in removing TrOCs from wastewater and develop a predictive model using the Solution Diffusion Model. Experiments were conducted using a stirred cell setup at various target concentrations, stirring speeds, and operating pressures, with acetaminophen and caffeine selected as representative pharmaceutical compounds. The results demonstrated that most of the pharmaceutical compounds were effectively removed, showing excellent performance. NF membranes exhibited high permeate flux with somewhat lower removal efficiency (average 84.17%), while RO membranes demonstrated high removal efficiency (average 99.21%), highlighting their importance in trace pharmaceutical treatment. The predictive model based on the solution diffusion model correlated well with the experimental data, suggesting its potential utility for large-scale system applications. This study confirms that NF and RO membranes are effective technologies for the removal of TrOCs from wastewater, offering a promising solution to the challenges posed by trace pharmaceutical contaminants.

## 1. Introduction

Trace organic contaminants (TrOCs), including pharmaceutically active compounds (PhACs), have emerged as critical environmental pollutants due to their persistence and potential toxicity [1]. These contaminants include a wide range of compounds such as pharmaceuticals and personal care products (PPCPs), steroids [2], endocrine-disrupting compounds (EDCs) [3], and pesticides [4]. The persistence of TrOCs is strongly related to their chemical structures, which often consist of electron-donating groups (EDGs) or electron-withdrawing groups (EWGs) [5]. Despite the advancements in wastewater treatment technologies, traditional treatment processes, which are mostly aerobic and anaerobic, have been shown to be ineffective in completely removing these contaminants [1,6].

PhACs, in particular, are of concern due to their low biodegradability and widespread detection in aquatic environments, even at low concentrations. This includes drugs like diclofenac, ibuprofen, and sulfamethoxazole, which have been found to cause ecotoxicological effects on aquatic organisms [7,8]. The continuous discharge of antibiotics into the environment also exacerbates the issue of antimicrobial resistance (AMR), posing a major threat to both ecosystems and public health [2].

To address this, advanced treatment technologies such as NF and RO membranes have gained prominence. These membrane technologies offer promising removal efficiencies for a wide variety of PhACs, with RO membranes showing up to 99% removal rates [6]. NF membranes, while offering lower energy consumption, generally show slightly lower removal rates but remain highly effective against larger molecular weight compounds [3]. Additionally, chemical cleaning processes for NF membranes have been shown to influence the rejection of PhACs, improving membrane longevity and performance [8,9].

Additionally, membrane filtration, other methods like advanced oxidation processes (AOPs), including ozone-activated peroxymonosulfate, have been used to degrade TrOCs in water, with promising results in eliminating these pollutants without producing harmful by-products [2,10,11,12,13]. As research continues, combining multiple treatment technologies, such as AOPs with NF or RO, is seen as a way to further enhance the removal efficiency of these persistent contaminants [14,15,16,17,18].

This study aims to treat PhACs using NF and RO technologies [14,19]. NF and RO are both effective in removing various substances through different mechanisms based on molecular size, hydrophobicity, and ionization [20,21]. NF, in particular, uses size exclusion mechanisms to filter out larger molecules, while ionized compounds are removed through electrostatic interactions [3,22,23,24]. On the other hand, RO can achieve near-complete removal of most PhACs, including smaller molecules and non-ionized compounds, by using high pressure and extremely tight membrane pores [6,23]. This research focuses on these removal mechanisms, evaluating the efficiency of NF and RO based on the physicochemical properties of the compounds, such as molecular weight, log *K_ow_* (hydrophobicity), and pKa (ionization state). The goal is to compare and analyze the effectiveness of these technologies across a variety of PhACs, ultimately recommending suitable treatment methods based on the specific properties of the contaminants [22].

In addition to the NF and RO removal experiments, the solution diffusion model (SDM) was applied to predict performance under various operational conditions [25]. SDM is a theoretical model that describes the transport of solute and solvent through a membrane, driven by concentration gradients. In this study, after analyzing the experimental results, SDM was employed to predict PhACs removal performance under different conditions [9]. The experimental data were compared with the SDM predictions, and an accurate predictive model was developed based on the findings. This model serves as a critical tool for designing PhACs removal processes applicable beyond experimental conditions, allowing for more precise predictions of NF and RO performance in real-world environments. By utilizing the experimental results to inform the SDM, this research aims to provide a deeper understanding of how NF and RO membranes function in various contexts, ensuring better removal efficiency under a range of operational scenarios.

## 2. Materials and Methods

### 2.1. Feed Solution

Experiments were conducted on 13 pharmaceutical compounds among various trace organic contaminants. All solvents used were of LC-MS grade, and all chemical reagents were of analytical grade. Methanol (J.T.Baker^®^, HPLC grade, 99.9%) was purchased from Avantor (Radnor, PA, USA), and ammonium fluoride (Merk, ACS, 99.99%) was obtained from Sigma-Aldrich (St. Louis, MO, USA). The 13 pharmaceutical reagents, including acetaminophen (ACP), caffeine (CFI), carbamazepine (CBZ), diclofenac (DCF), ibuprofen (IBF), iopromide (IPM), lincomycin (LCM), naproxen (NPX), propranolol (PPN), ranitidine (RNT), sulfamethoxazole (SFX), sulfamethazine (SFA), and trimethoprim (TMP), were also purchased from Sigma-Aldrich (St. Louis, MO, USA).

Stock solutions of all compounds were prepared in methanol at a concentration of 1000 mg/L. The deionized water used in the experiments was produced using a purification system and had a resistivity of 18.2 MΩ·cm at 25 °C. The chemical formulas and molecular weights of each compound were summarized in Table 1. Samples for the experiments were prepared by quantitatively dissolving each compound in a NaCl 500 mg/L aqueous solution at concentrations of 50 ng/L. The pH of all aqueous solutions containing trace amounts of PhACs was consistently 6.7.

### 2.2. Membrane Filtration

**Figure 1:**
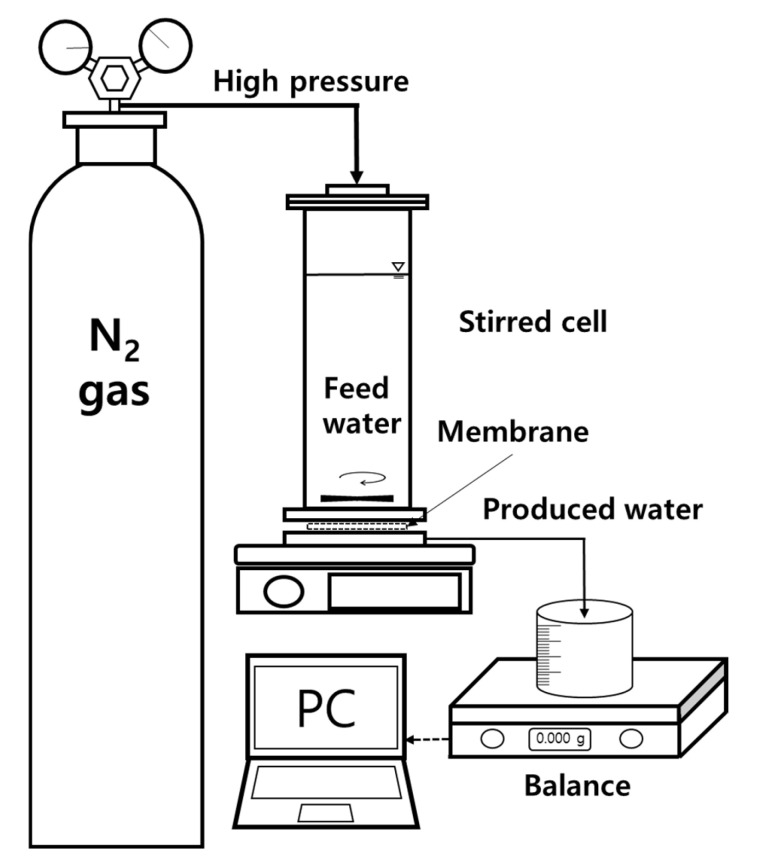
Schematic diagram of the stirred cell module.

In this study, experiments were performed to assess the removal efficiency of various trace organic contaminants (TrOCs) using NF and RO membranes. The NF membrane utilized was NF90 (FilmTec™, Minneapolis, MN, USA), with a pore size of approximately 0.2–0.5 nm and composed of a polyamide thin-film composite. The RO membrane used was SWC6-4040 (Nitto Denko Corp., Osaka, Japan), designed for seawater desalination. It is a dense, polyamide thin-film composite membrane, where separation occurs via the solution diffusion mechanism, rather than through pores. Prior to the experiments, all membranes were activated by soaking them in distilled water for 24 h. The membrane module employed was the HP4750 Stirred cell from Sterlitech (Auburn, WA, USA), featuring an effective membrane area of 14.6 cm^2^ and a membrane thickness of 200 μm. The experimental setup, as illustrated in Figure 1, was configured with an initial feed volume of 300 mL. The experiments were carried out at an operating pressure of 10 bar, with stirring speeds of 300 rpm and 450 rpm, and the temperature was kept constant at 25 ± 1 °C. The two rpm (stirring speed) were chosen to control concentration polarization by increasing surface velocity at the membrane interface. In typical membrane separation mechanisms, concentration polarization significantly impacts performance, often leading to membrane fouling. However, the removal of PhACs in this study was influenced not only by basic separation mechanisms but also by the specific physicochemical properties of each compound. This aspect was incorporated into the SDM, and different RPM conditions were utilized to derive parameters for the PhACs removal prediction model. Permeate flux was continuously monitored using a balance connected to a computer. The volume concentration factor (VCF) is defined as the ratio of the initial feed volume to the concentrated volume and is calculated as follows:

(1) $$VCF=\frac{V_{f}}{V_{f}-V_{p}}$$

where, $V_{f}$ is the initial feed volume, and $V_{p}$ is the permeate volume. The initial feed solution was prepared by dissolving 13 different TrOCs in a NaCl 500 mg/L aqueous solution at concentrations of 50 ng/L for each compound. Permeate samples were collected at regular intervals to measure flux, and the TrOCs concentrations in the permeate were analyzed using LC-MS. The collected data were then used to calculate the removal efficiency and flux under each experimental condition.

### 2.3. Analysis of Water Quality

The LC–MS/MS analysis was performed on a Vanquish High-Performance Liquid Chromatography (HPLC) system (Thermo, Waltham, MA, USA) coupled with a TSQ Quantis™ Triple Quadrupole Mass Spectrometer (Thermo, Waltham, MA, USA) equipped with an electrospray ionization (ESI) interface in multiple-reaction monitoring (MRM) mode. Nitrogen (purity, 99.9%) gas was used. LC and MS parameters are summarized in Table 2.

### 2.4. Solution Diffusion Model

The solution diffusion model is a widely recognized framework for describing the mechanism by which solutes move through membranes. This model assumes that solutes are transported through the membrane matrix by diffusion, driven by concentration gradients [25,26]. In this study, the SDM, which considers concentration polarization, was applied to analyze the experimental results and predict removal performance under various operating conditions. According to the SDM, the transport of solutes and solvents occurs independently, with solute transport being driven by the concentration difference across the membrane [27,28]. Furthermore, in addition to the basic removal mechanisms of the membrane, specific characteristics of PhACs, such as hydrophobicity and ionization tendency, were used to derive $L_{s}$ values from each experiment [29]. Incorporating these $L_{s}$ values aims to enhance the accuracy of the SDM in predicting PhACs removal rates beyond simple salt rejection. This approach allows for more reliable predictions of PhACs removal performance, accommodating the unique physicochemical properties of these compounds, which affect their interaction with the membrane.

The solute flux $J_{s}$ is expressed as the product of the solute permeability coefficient $L_{s}$ and the difference between the feed concentration $C_{b}$ and the permeate concentration $C_{p}$ [28]:

(2) $$J_{s}=L_{s}\;(C_{b}-C_{p})$$

The solvent flux $J_{v}$ is determined by the membrane water permeability $L_{v}$, the pressure difference ∆P, and the osmotic pressure difference ∆π:

(3) $$J_{v}=L_{v}(∆P-∆\pi )$$

$L_{v}$ is the solvent transport parameter, $L_{s}$ is the solute transport parameter, $C_{b}$ is the solute concentration in the bulk solution, $C_{p}$ is the solute concentration on the permeate side, ∆π is the osmotic pressure, and ∆P is the transmembrane pressure. However, as filtration progresses, concentration polarization occurs at the membrane surface, so the equations at the surface need to be modified as follows.

(4) $$J_{s}=L_{s}\;\left(C_{m}-C_{p}\right)$$

$C_{m}$ is the solute concentration at the membrane surface, and it is calculated according to the following film theory. The concentration profile at the surface is calculated using the following equation [28].

(5) $$\frac{C_{m}-C_{p}}{C_{b}-C_{p}}=e^{\frac{J_{v}}{k}}$$

where *k* is the mass transfer coefficient for the back diffusion of the bulk solution from the membrane on the high-pressure side, and the growth of the concentration boundary layer is suppressed by stirring. The mass transfer coefficient k follows the equation below.

(6) $$k={0.104(\frac{D_{sw}}{r})(\frac{wr^{2}\rho }{\mu })}^{\frac{2}{3}}{\left(\frac{\mu }{{\rho D}_{sw}}\right)}^{\frac{1}{3}}$$

where, $D_{sw}$ is the solute diffusion coefficient, w is the stirring speed, r is the stirring radius, μ is the viscosity of the liquid at 25 °C, and ρ represents the density of the solution.

## 3. Results and Discussion

### 3.1. PhAC Removal Performance and Flux Analysis in NF Membranes

**Figure 2:**
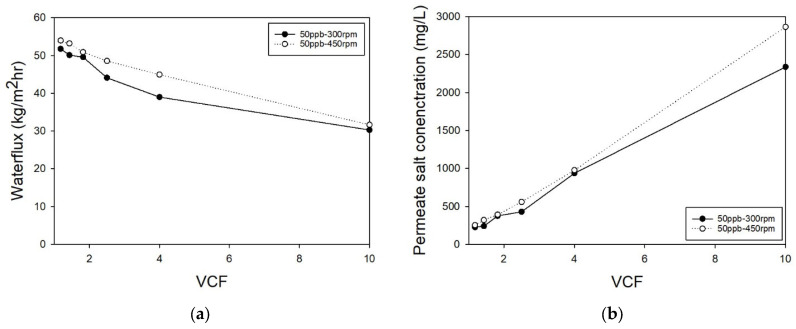
The result of NF experiment. (**a**) Permeate water flux and (**b**) NaCl concentration of permeate by VCF.

**Figure 3:**
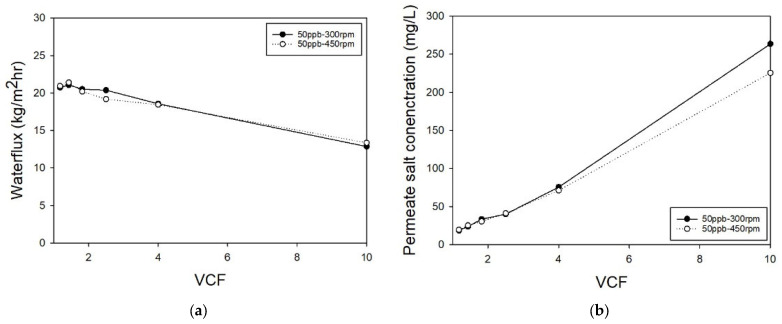
The result of RO experiment. (**a**) Permeante waterflux and (**b**) NaCl concentration of permeate by VCF.

The experimental results for PhAC removal using NF membranes are presented in Figure 1. For each Volume Concentration Factor (VCF), water flux [kg/m^2^·h, LMH], NaCl concentration [mg/L], and target concentration [mg/L] were measured with every 30 mL of permeate produced. As the experiment progressed, flux decreased due to increased osmotic pressure from feed concentration, rather than membrane fouling. Higher stirring speeds, from 300 rpm to 450 rpm, improved both flux and removal efficiency by reducing concentration polarization. Figure 2 and Figure 3 represent results at 300 rpm and 450 rpm, respectively, and helped derive $L_{S}$ and $L_{v}$ values for the solution diffusion model. The presence of 50 ng/L of pharmaceuticals had minimal impact on flux reduction, instead, flux decline was mainly attributed to the increase in osmotic pressure caused by the concentrated NaCl within the stirred cell.

Table 3 shows their average removal rates. The NF treatment achieved removal rates between 77% and 94% for PhACs, with molecular weight being a significant factor. Larger molecules, like Iopromide (791.12 g/mol), showed a higher removal rate (92.8%), while smaller ones, such as Acetaminophen (151.16 g/mol), had lower rates. However, removal was not strictly proportional to molecular weight due to ionization effects. For example, Diclofenac (pKa 4) and Ibuprofen (pKa 4.9) were ionized in neutral or acidic conditions, leading to higher removal rates (86.05% and 87.88%). Caffeine, with a higher pKa, remained mostly non-ionized, so its removal was influenced more by molecular size and hydrophobicity. Hydrophobicity, indicated by log *K_ow_*, also impacted removal efficiency. Compounds with higher log *K_ow_* values, like diclofenac (4.51) and ibuprofen (3.97), showed better removal rates, while substances with lower log *K_ow_*, such as caffeine (0.07), exhibited lower rates (85.07%).

The relative differences in removal efficiency due to the properties of the three factors (molecular weight, ionization, and hydrophobicity) were observed for each substance [3,6,20,21]. The characteristics of the 13 compounds are as follows:

Acetaminophen, with its small molecular weight and low log *K_ow_*, is highly hydrophilic, allowing it to pass easily through the NF membrane, resulting in a lower removal rate. Its high pKa also means it remains mostly non-ionized, minimizing electrostatic interactions and reducing removal efficiency. Caffeine is very hydrophilic and non-ionized, which leads to lower removal rates in NF membranes, as its small size allows it to permeate easily. Carbamazepine has moderate hydrophobicity, leading to higher removal rates in NF, as its larger molecular size makes it more difficult to permeate the membrane. Despite being mostly non-ionized, its hydrophobicity increases removal efficiency. Diclofenac is highly hydrophobic and negatively charged above pH 4, resulting in high removal efficiency in NF due to its large molecular weight and electrostatic interactions. Ibuprofen exhibits strong hydrophobicity and ionizes at pH levels above 5, allowing for effective removal in NF, supported by its relatively large molecular weight. Iopromide has a very large molecular weight, limiting its permeation through NF membranes and leading to high removal efficiency, despite its moderate hydrophobicity. Lincomycin is highly hydrophilic and ionizes at neutral pH, resulting in lower removal rates. Despite its large molecular size, its hydrophilicity reduces its NF removal efficiency. Naproxen is hydrophobic and carries a negative charge above pH 4, resulting in high NF removal efficiency, especially with its large molecular size. Propranolol is hydrophobic but non-ionized at pH 7, leading to lower removal rates due to limited electrostatic interactions. Ranitidine is mostly non-ionized, with low electrostatic interactions and hydrophobicity, resulting in lower removal rates despite its large molecular weight. Sulfamethoxazole becomes negatively charged at pH 7, increasing its removal via electrostatic interactions, while its larger molecular size enhances size exclusion. Sulfamethazine is hydrophilic and ionizes due to its pKa, allowing for moderate removal in NF based on ionization and molecular size. Trimethoprim is non-ionized and highly hydrophilic at neutral pH, leading to lower removal efficiency in NF. In NF membranes, the removal efficiency for pharmaceutically active compounds (PhACs) is influenced by multiple factors beyond the basic membrane mechanisms of size exclusion and charge exclusion. While higher removal rates are typically associated with larger molecular weights, the specific physicochemical properties of each compound—such as molecular weight, hydrophobicity (log *K_ow_*), and ionization state (pKa)—collectively play substantial roles in determining removal efficiency. Compounds that are highly hydrophobic or ionized, like Diclofenac and Naproxen, tend to exhibit higher removal rates due to enhanced interactions with the membrane surface. Conversely, highly hydrophilic and non-ionized compounds, such as Acetaminophen and Caffeine, pass through the NF membrane more readily, resulting in lower removal efficiencies.

For certain compounds, these intrinsic physicochemical properties limit the impact of increased stirring speed (rpm) on removal rates. For example, Acetaminophen, with its low molecular weight, high hydrophilicity, and minimal ionization, permeates the NF membrane easily, leading to lower removal rates even under conditions of elevated rpm. Similarly, Caffeine’s high hydrophilicity and non-ionized state result in minimal enhancement in removal rates with increased rpm, as its small molecular size allows it to traverse the membrane with ease. In contrast, compounds like Carbamazepine, with moderate hydrophobicity and larger molecular size, show improved removal efficiency due to the additional resistance posed by these characteristics, which inhibit easy permeation. Likewise, highly hydrophobic and negatively charged compounds like Diclofenac and Ibuprofen exhibit enhanced removal efficiency due to both electrostatic interactions and size exclusion mechanisms. For these compounds, the combined effects of increased hydrophobicity and charge contribute to higher removal efficiencies.

This pattern indicates that the performance of NF membranes for PhAC removal cannot be solely attributed to molecular size. Rather, the interplay among molecular weight, ionization, and hydrophobicity drives the observed variability in removal efficiency, underscoring that size exclusion is only one component of a more complex rejection mechanism. In sum, the findings demonstrate that increased rpm has a limited effect on the removal rates of highly hydrophilic or non-ionized compounds, as these properties inherently facilitate easier passage through the NF membrane, whereas compounds with higher hydrophobicity or significant ionization experience enhanced rejection rates across operational settings.

### 3.2. PhAC Removal Performance and Flux Analysis in RO Membranes

In the RO experiment, very high removal rates for various trace organic compounds (TrOCs) were observed. RO demonstrated exceptional performance, particularly with large molecular weight and hydrophobic compounds, with most substances achieving removal rates above 90%. For example, iopromide, with a large molecular weight, showed over 99% removal, attributed to RO’s small pore size and high pressure. Similarly, hydrophobic substances like Diclofenac and Ibuprofen also had removal rates above 98%. Table 4 provides a summary of the rejection rates for selected pharmaceutically active compounds (PhACs) under two different stirring speeds, showcasing consistently high removal efficiencies across all tested compounds. However, specific compounds with lower molecular weights and high hydrophilicity, such as acetaminophen and caffeine, exhibited minimal additional improvement in removal with increased stirring speeds (450 rpm). This outcome suggests that for certain hydrophilic TrOCs, enhanced stirring may facilitate permeability rather than improve rejection by reducing concentration polarization. These nuances in removal efficiency highlight the interplay between molecular characteristics and operational conditions in RO filtration. RO effectively removed both ionized and non-ionized substances, outperforming traditional water treatment processes. Furthermore, RO performed consistently across different pH conditions, indicating that its removal mechanism is primarily driven by pore size and pressure differences rather than electrostatic interactions. The results confirmed that RO provides high removal efficiency regardless of molecular weight, hydrophobicity, or ionization state, showing superior performance compared to NF. Figure 3 illustrates the operating flux of the RO system, with $L_{s}$ and $L_{v}$ values calculated based on water flux, salt rejection, and the removal efficiency of different compounds. Similar to the NF findings, pharmaceuticals at a concentration of 50 ng/L had a negligible effect on flux reduction, with flux decline predominantly driven by the rise in osmotic pressure from accumulated NaCl within the stirred cell. These derived values were then applied to the solution diffusion model to predict salt rejection rates under conditions beyond those tested, further confirming the versatility of RO membranes in removing a broad range of PhACs under various operating conditions and ensuring robust and reliable removal performance.

In this study, the SDM was applied to evaluate PhACs removal performance under low-pressure conditions. Although high salt concentrations typically require higher operational pressure, this experiment was conducted at low salinity levels. For this reason, a seawater RO membrane was selected, as it provides a clearer comparison to NF membranes in terms of PhAC removal, enabling a more direct and informative evaluation for readers. This choice also offers insights into the performance of SWRO membranes under low salinity conditions, extending the applicability of SDM-based predictions to a broader range of conditions.

### 3.3. Predicting Removal Efficiency Using the Solution Diffusion Model

Using the $L_{s}$ values derived from NF and RO experiments, the solution diffusion model was applied to predict the removal rates of target compounds under various operating conditions, such as pressure and stirring speed. The Ls values for each compound, derived from the experimental results, are presented in Table 5. $L_{s}$ values, representing solute transport parameters, helped estimate how these parameters affected removal efficiency. Additionally, the root-mean-square error (RMSE) was utilized to validate the accuracy of the model’s predictions against experimental data [30,31]. By incorporating RMSE, the model provided more precise insights, showing how adjustments in pressure or stirring speed could optimize NF and RO membrane performance under different conditions.

RMSE (Root Mean Square Error) is a crucial metric in the solution diffusion model (SDM) used to assess the difference between predicted and actual experimental results. It evaluates the model’s prediction accuracy, with a smaller RMSE indicating a closer fit between the model’s predictions and actual data, while a larger RMSE signifies greater prediction errors. The Root Mean Square Error is calculated using the following equation:

(7) $$RMSE=\sqrt{\frac{1}{n}\sum_{i=1}^{n}{\left(y_{i,pred}-y_{i,true}\right)}^{2}}$$

where, $y_{i,pred}$ represents the predicted values, $y_{i,true}$ denotes the actual measured values, and *n* is the number of data points. RMSE provides a measure of the average magnitude of errors between predicted and actual values, with lower RMSE values reflecting better model accuracy. In practical applications, RMSE plays a vital role in determining how well the SDM fits the experimental data and the model’s overall reliability. A lower RMSE indicates minimal error, suggesting that the model is effectively predicting system behavior. This metric is critical for evaluating the SDM’s performance, enabling refinements and validation to improve its predictive accuracy. Particularly in membrane filtration systems such as NF and RO, where precise modeling of solute transport is essential, RMSE serves as a key indicator of system optimization. By providing a single numerical value, RMSE offers a straightforward means to compare different models or modifications within a single model, ultimately enhancing the understanding of the system and allowing for more accurate predictions across varying operational conditions.

**Figure 4:**
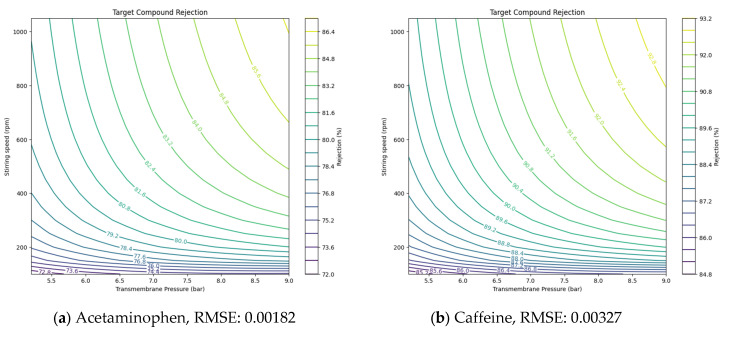

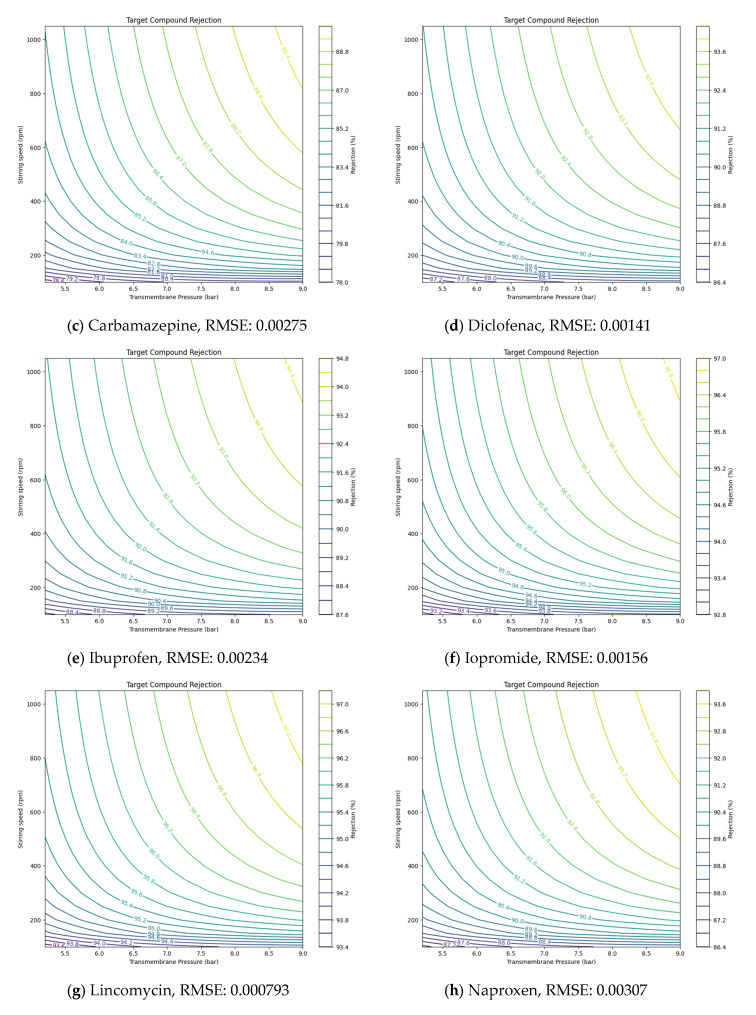

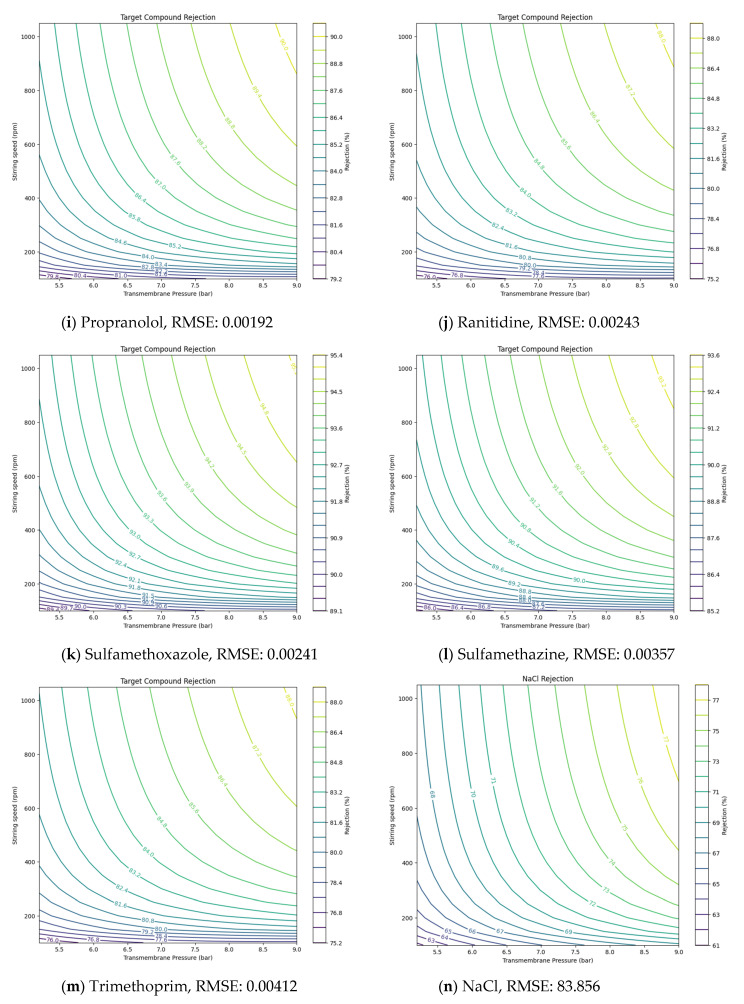
Prediction of PhACs removal efficiency and RMSE value in NF membranes based on the solution diffusion model (**a**–**n**).

**Figure 5:**
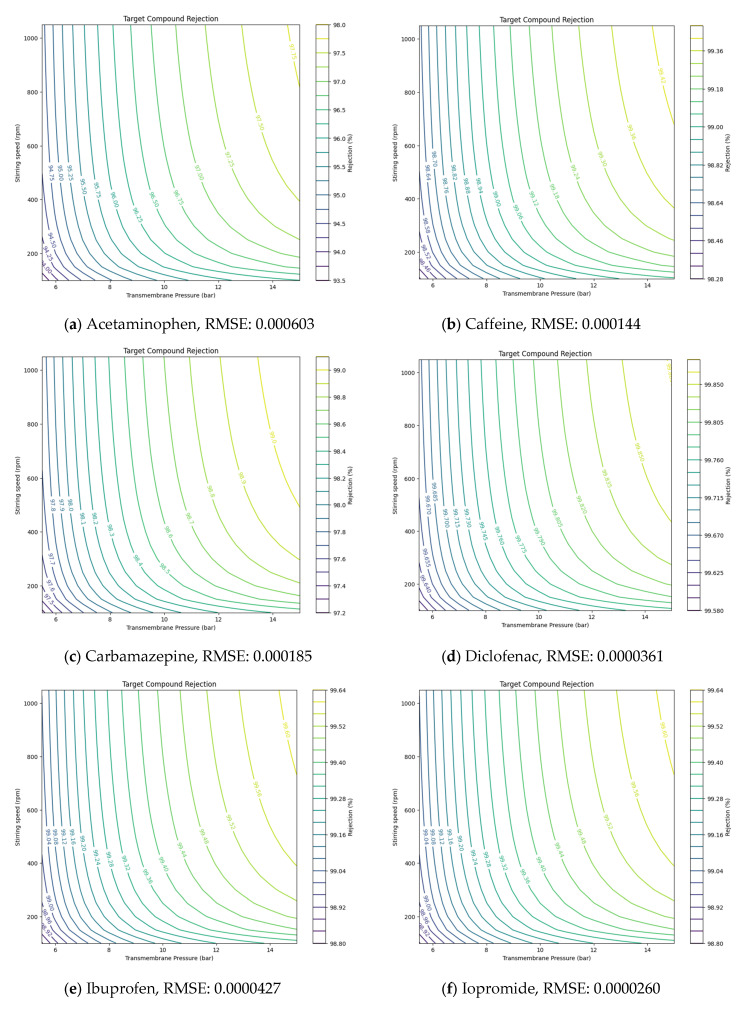

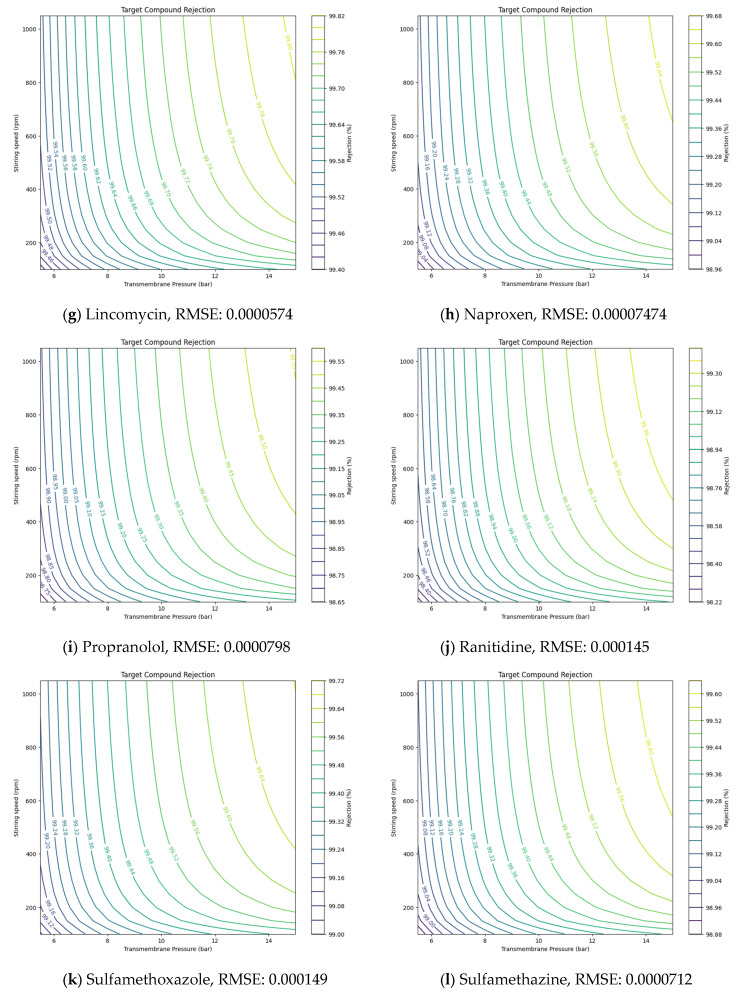

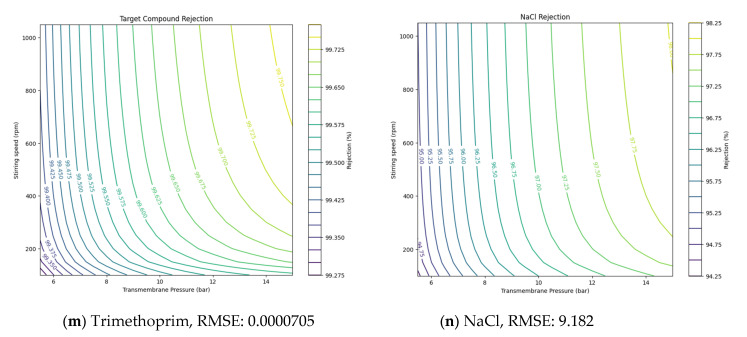
Prediction of PhACs removal efficiency and RMSE value in RO membranes based on the solution diffusion model (**a**–**n**).

After numerically evaluating the model’s performance, the following graphs compare the actual removal rates with the predicted values for each compound. Figure 4 and Figure 5 visually demonstrate how accurately the solution diffusion model performed under various operational conditions. In Figure 4, the graph is limited to 9 bar as removal efficiency showed greater variation with rpm under conditions of 9 bar and below, while the effect of lower rpm was minimal at pressures above 10 bar. This comparison allows for a clear understanding of the model’s prediction accuracy and provides deeper insight into the system’s performance under different conditions.

This demonstrates that the solution diffusion model is capable of accurately predicting removal rates across both tested and untested operational conditions. The findings highlight the model’s strength and dependability, underscoring its potential for broader application in forecasting removal efficiency under various environmental scenarios.

### 3.4. Validation of the Solution Diffusion Model for Removal Efficiency Prediction

In this study, a Solution Diffusion Model was developed based on permeability coefficients (*L*_*s*_) derived from experimental results to predict the removal efficiency of various compounds. To verify the accuracy of this model, validation experiments were conducted under four randomly selected conditions. These validation experiments were performed under the same environmental settings (equipment, temperature, pH, etc.) as the primary experiments, with only the operating pressure and stirring speed varied. The experiments conducted for each model were based on the *L*_*s*_ values calculated according to the molecular size and the physical properties (ionization and hydrophilicity) of each compound, enabling effective prediction of the removal efficiency of the 13 pharmaceutical compounds tested with NF and RO membranes. As summarized in Table 6, validation experiments for the four selected compounds (Acetaminophen, Iopromide, Sulfamethazine, and Caffeine) under random conditions confirmed the prediction accuracy of the SDM model. As shown in Table 6, the predicted and observed rejection rates across all experimental conditions were found to be highly consistent, with each validation experiment yielding high accuracy within the RMSE range. For instance, in the case of NF membranes, the predicted rejection rate of Acetaminophen was 76.42% at 8 bar and 250 rpm, while Iopromide achieved a rejection rate of 95.69% at 6 bar and 500 rpm. For RO membranes, the rejection rates of Sulfamethazine and Caffeine were 99.28% at 9 bar and 200 rpm and 99.12% at 12 bar and 500 rpm, respectively. It demonstrates that the SDM model developed in this study provides consistent predictive accuracy under various operational conditions.

## 4. Conclusions

This study experimentally demonstrated the effectiveness of NF and RO membranes in removing pharmaceutically active compounds (PhACs) from wastewater, providing foundational insights into the optimization of membrane-based water treatment systems. The results confirmed that membrane type and structure, as well as compound-specific characteristics—such as molecular weight, hydrophobicity, and ionization state—are key factors influencing removal efficiency. RO generally outperformed NF due to its denser structure, which is more effective for smaller and non-ionized compounds. By incorporating these characteristics, permeability coefficients (*L*_*s*_) were calculated for each compound, supporting the development of a tailored solution diffusion model that accurately reflects the behavior of diverse PhACs across varying conditions.

The SDM, validated through low RMSE values across four distinct experimental settings, exhibited reliable predictive performance under both tested and untested scenarios, offering a robust tool for optimizing NF and RO systems for PhAC removal. This validated model not only provides insights into the removal processes of complex pollutants under diverse water conditions but also underscores the potential for further model-based optimizations in real-world water treatment applications.

In summary, this research significantly contributes to advancing membrane-based water treatment solutions capable of efficiently removing PhACs from wastewater, offering valuable data and a predictive framework to enhance the applicability of NF and RO technologies in managing complex environmental contaminants.

## Figures and Tables

**Table 1:** Physicochemical properties of pharmaceutically active compounds (PhACs).

PhACs	Chemical Formula	Molecular Weight (g/mol)	log *K_ow_*	pKa
Acetaminophen	C_8_H_9_NO_2_	151.16	0.46	9.5
Caffeine	C_8_H_10_N_4_O_2_	194.19	−0.07	14
Carbamazepine	C_15_H_12_N_2_O	236.27	2.45	13.9
Diclofenac	C_14_H_11_C_l2_NO_2_	296.15	4.51	4.0
Ibuprofen	C_13_H_18_O_2_	206.28	3.97	4.9
Iopromide	C_18_H_24_I_3_N_3_O_8_	791.12	2.60	3.4
Lincomycin	C_18_H_34_N_2_O_6_S	406.53	−0.69	7.6
Naproxen	C_14_H_14_O_3_	230.26	3.18	4.15
Propranolol	C_16_H_21_NO_2_	259.34	3.48	9.5
Ranitidine	C_13_H_22_N_4_O_3_S	314.41	0.24	8.2
Sulfamethoxazole	C_10_H_11_N_3_O_3_S	253.28	0.89	5.6
Sulfamethazine	C_12_H_14_N_4_O_2_S	278.33	0.14	7.4
Trimethoprim	C_14_H_18_N_4_O_3_	290.32	0.91	7.2

**Table 2:** LC and MS parameters.

Instrument	Conditions								
LC Condition	Mobile phase	A: 0.1 mM ammonium fluoride in water, B: Methanol							
	Gradient program	Time (min)	0	1	10	21	25	25.5	30
		B (%)	5	20	100	100	5	5	5
	Flow rate	0.3 mL/min							
	Column	Hypersil GOLD C18 (2.1 mm × 100 mm × 1.9 μm)							
	Column temperature	45 °C							
	Injection volume	10 μL							
MS Condition	Ionization Source	Electrospray ionization (ESI)							
	Positive Ion Spray Voltage	3800 V							
	Negative Ion Spray Voltage	3500 V							
	Ion Transfer Tube Temperature	325 °C							
	Sheath gas	50 Arb							
	Aux gas	10 Arb							
	Sweep Gas	1 Arb							
	Vaporizer Temperature	350 °C							

**Table 3:** Rejection of PhACs in NF membranes.

PhACs	Rejection (%)	
	300 rpm	450 rpm
Acetaminophen	77.87	76.55
Caffeine	85.07	84.59
Carbamazepine	82.10	77.73
Diclofenac	86.06	89.58
Ibuprofen	87.88	89.20
Iopromide	92.80	93.23
Lincomycin	84.52	85.37
Naproxen	86.02	88.61
Propranolol	78.96	78.90
Ranitidine	77.03	75.60
Sulfamethoxazole	91.42	88.74
Sulfamethazine	85.76	87.73
Trimethoprim	77.12	79.87

**Table 4:** Rejection of PhACs in RO membranes.

PhACs	Rejection (%)	
	300 rpm	450 rpm
Acetaminophen	96.36	96.42
Caffeine	99.23	99.28
Carbamazepine	98.66	98.75
Diclofenac	99.82	99.87
Ibuprofen	99.37	99.46
Iopromide	99.85	99.85
Lincomycin	99.72	99.73
Naproxen	99.50	99.45
Propranolol	99.37	99.25
Ranitidine	99.19	99.30
Sulfamethoxazole	99.50	99.54
Sulfamethazine	99.43	99.38
Trimethoprim	99.68	99.60

**Table 5:** $L_{s}$ values for each compound based on experimental results.

Target	$L_{s}$	
	NF Membrane (×10^−6^)	RO Membrane (×10^−6^)
Salt	3.492	0.170
Acetaminophen	1.745	0.178
Caffeine	0.838	0.044
Carbamazepine	1.281	0.073
Diclofenac	0.718	0.011
Ibuprofen	0.653	0.031
Iopromide	0.365	0.008
Lincomycin	0.335	0.015
Naproxen	0.725	0.027
Propranolol	1.212	0.035
Ranitidine	1.490	0.046
Sulfamethoxazole	0.575	0.025
Sulfamethazine	0.797	0.029
Trimethoprim	1.501	0.019

**Table 6:** Validation conditions and predicted rejection rates for selected PhACs under NF and RO membranes.

	NF		RO	
PhACs	Acetaminophen	Iopromide	Sulfamethazine	Caffeine
Operation pressure (bar)	8	6	9	12
Stirring speed (rpm)	250	500	200	500
Rejection (%)	76.42	95.69	99.18	99.21

## Data Availability

The original contributions presented in the study are included in the article, further inquiries can be directed to the corresponding author.

## References

[1] Alhalabi A.M., Meetani M.A., Shabib A., Maraqa M.A. (2024). Sorption of pharmaceutically active compounds to soils: A review. Environ. Sci. Eur..

[2] Azizi-Lalabadi M., Pirsaheb M. (2021). Investigation of steroid hormone residues in fish: A systematic review. Process Saf. Environ. Prot..

[3] Czech B., Rubinowska K. (2013). TiO_2_-assisted photocatalytic degradation of diclofenac, metoprolol, estrone and chloramphenicol as endocrine disruptors in water. Absorption.

[4] Tang Y., Long X., Wu M., Yang S., Gao N., Xu B., Dutta S. (2020). Bibliometric review of research trends on disinfection by-products in drinking water during 1975–2018. Sep. Purif. Technol..

[5] Tröger R., Wiberg K. (2018). Micropollutants in drinking water from source to tap—Method development and application of a multiresidue screening method. Sci. Total Environ..

[6] Deniere E., Demeestere K. (2023). The ozone-activated peroxymonosulfate process for the removal of a mixture of TrOCs with different ozone reactivity at environmentally relevant conditions: Technical performance, radical exposure and online monitoring by spectral surrogate parameters. J. Chem. Eng..

[7] Verliefde A., van Dijk E. (2008). Rejection of trace organic pollutants with high pressure membranes (NF/RO). Environ. Prog..

[8] Zhang Y., Zhu H. (2022). Insights into the penetration of PhACs in TCM during ultrafiltration: Effects of fouling mechanisms and intermolecular interactions. Sep. Purif. Technol..

[9] Simon A., Price W.E., Nghiem L.D. (2012). Effects of chemical cleaning on the nanofiltration of pharmaceutically active compounds (PhACs). Sep. Purif. Technol..

[10] Phan H.V., Hai F.I. (2014). Simultaneous nitrification/denitrification and trace organic contaminant (TrOC) removal by an anoxic–aerobic membrane bioreactor (MBR). Bioresour. Technol..

[11] Taheran M., Valero J.R. (2016). Membrane processes for removal of pharmaceutically active compounds (PhACs) from water and wastewaters. Sci. Total Environ..

[12] Zhou Z., Shao S. (2023). Effect of urea-based chemical cleaning on TrOCs rejection by nanofiltration membranes. Sep. Purif. Technol..

[13] Gavrilescu M., Fava F. (2015). Emerging pollutants in the environment: Present and future challenges in biomonitoring, ecological risks and bioremediation. New Biotechnol..

[14] Wang Y., Chen G. (2018). Removal of Pharmaceutical and Personal Care Products (PPCPs) from Municipal Waste Water with Integrated Membrane Systems, MBR-RO/NF. Int. J. Environ. Res. Public Health.

[15] Wijekoon K.C., Nghiem L.D. (2014). Rejection and fate of trace organic compounds (TrOCs) during membrane distillation. J. Membr. Sci..

[16] Alokpa K., Lafortune F., Cabana H. (2022). Application of laccase and hydrolases for trace organic contaminants removal from contaminated water. Environ. Adv..

[17] Li X., Zhang X. (2022). Application of sulfate radicals-based advanced oxidation technology in degradation of trace organic contaminants (TrOCs): Recent advances and prospects. J. Environ. Manag..

[18] Ol A., Omotola E.O., Olatunji O.S. (2020). Pharmaceuticals and personal care products in water and wastewater: A review of treatment processes and use of photocatalyst immobilized on functionalized carbon in AOP degradation. BMC Chem..

[19] Mahlangu O.T., Nthunya L.N., Motsa M.M., Richards H., Mamba B.B., Shah M.P. (2023). Treatment of trace organics and emerging contaminants using traditional and advanced technologies. Industrial Wastewater Reuse.

[20] Comerton A.M., Andrews R.C. (2008). The rejection of endocrine disrupting and pharmaceutically active compounds by NF and RO membranes as a function of compound and water matrix properties. J. Membr. Sci..

[21] Maryam B., Buyukgungor H. (2020). A study on behavior, interaction and rejection of paracetamol, diclofenac and ibuprofen (PhACs) from wastewater by nanofiltration membranes. Environ. Technol. Innov..

[22] Giacobbo A., Pinho M.N. (2023). Ultrafiltration and Nanofiltration for the removal of pharmaceutically active compounds from water: The effect of operating pressure on electrostatic solute—Membrane interactions. Membranes.

[23] Zhao Y., Taylor J.S. (2005). Predicting RO/NF water quality by modified solution diffusion model and artificial neural networks. J. Membr. Sci..

[24] Li C., Yang Y. (2018). Removal of PhACs and their impacts on membrane fouling in NF/RO membrane filtration of various matrices. J. Membr. Sci..

[25] Heiranian M., Elimelech M. (2023). Mechanisms and models for water transport in reverse osmosis membranes: History, critical assessment, and recent developments. Chem. Soc. Rev..

[26] Bhinder A., Shabani S., Sadrzadeh M. (2018). Effect of Internal and External Concentration Polarizations on the Performance of Forward Osmosis Process. Osmotically Driven Membrane Processes—Approach, Development and Current Status.

[27] Ghazali N.F., Lim K. (2020). Mass Transport Models in Organic Solvent Nanofiltration: A Review. J. Adv. Res. Fluid. Mech. Therm. Sci..

[28] Choi Y., Oh H., Lee S., Choi Y., Hwang T.M., Jeon J.C., Choung Y.K. (2010). Removal of Taste and Odor Model Compounds (2-MIB and Geosmin) with the NF Membrane. Desalination Water Treat..

[29] Oh H., Hwang T., Lee S. (2009). A simplified simulation model of RO systems for seawater desalination. Desalination.

[30] Monroe J.I., Thamaraiselvan C., Wickramasinghe S.R. (2024). Grand challenges in membrane transport, modeling and simulation. Front. Membr. Sci..

[31] Hodson T.O. (2022). Root-mean-square error (RMSE) or mean absolute error (MAE): When to use them or not. Geosci. Model. Dev..

